# RNA-seq of nine canine prostate cancer cell lines reveals diverse therapeutic target signatures

**DOI:** 10.1186/s12935-021-02422-9

**Published:** 2022-02-02

**Authors:** Eva-Maria Packeiser, Leila Taher, Weibo Kong, Mathias Ernst, Julia Beck, Marion Hewicker-Trautwein, Bertram Brenig, Ekkehard Schütz, Hugo Murua Escobar, Ingo Nolte

**Affiliations:** 1 Small Animal Clinic, University of Veterinary Medicine Hannover, Hannover, Germany; 2 Department of Medicine, Clinic III, Hematology, Oncology and Palliative Medicine, University Medical Center Rostock, Rostock, Germany; 3 Institute of Biomedical Informatics, Graz University of Technology, Graz, Austria; 4 Division of Bioinformatics, Department of Biology, Friedrich-Alexander Universität Erlangen-Nürnberg (FAU), Erlangen, Germany; 5 Chronix Biomedical, Göttingen, Germany; 6 Department of Pathology, University of Veterinary Medicine Hannover, Hannover, Germany; 7 University of Göttingen, Institute of Veterinary Medicine, Göttingen, Germany; 8 Comprehensive Cancer Center Mecklenburg-Vorpommern (CCC-MV), Campus Rostock, University of Rostock, 18057 Rostock, Germany; 9 Institute for Biostatistics and Informatics in Medicine and Ageing Research, Rostock University Medical Center, 18057 Rostock, Germany; 10 Institute of Muscle Biology and Growth, Research Institute for Farm Animal Biology (FBN), 18196 Dummerstorf, Germany

**Keywords:** Prostate cancer, Metastasis, Bladder cancer, TCC, Cell line, Dog, Gene expression, In vitro model, Targeted therapy

## Abstract

**Background:**

Canine prostate adenocarcinoma (PAC) and transitional cell carcinoma (TCC) are typically characterized by metastasis and chemoresistance. Cell lines are important model systems for developing new therapeutic strategies. However, as they adapt to culturing conditions and undergo clonal selection, they can diverge from the tissue from which they were originally derived. Therefore, a comprehensive characterization of cell lines and their original tissues is paramount.

**Methods:**

This study compared the transcriptomes of nine canine cell lines derived from PAC, PAC metastasis and TCC to their respective original primary tumor or metastasis tissues. Special interests were laid on cell culture-related differences, epithelial to mesenchymal transition (EMT), the prostate and bladder cancer pathways, therapeutic targets in the PI3K-AKT signaling pathway and genes correlated with chemoresistance towards doxorubicin and carboplatin.

**Results:**

Independent analyses for PAC, PAC metastasis and TCC revealed 1743, 3941 and 463 genes, respectively, differentially expressed in the cell lines relative to their original tissues (DEGs). While genes associated with tumor microenvironment were mostly downregulated in the cell lines, patient-specific EMT features were conserved. Furthermore, examination of the prostate and bladder cancer pathways revealed extensive concordance between cell lines and tissues. Interestingly, all cell lines preserved downstream PI3K-AKT signaling, but each featured a unique therapeutic target signature. Additionally, resistance towards doxorubicin was associated with G2/M cell cycle transition and cell membrane biosynthesis, while carboplatin resistance correlated with histone, m- and tRNA processing.

**Conclusion:**

Comparative whole-transcriptome profiling of cell lines and their original tissues identifies models with conserved therapeutic target expression. Moreover, it is useful for selecting suitable negative controls, i.e., cell lines lacking therapeutic target expression, increasing the transfer efficiency from in vitro to primary neoplasias for new therapeutic protocols. In summary, the dataset presented here constitutes a rich resource for canine prostate and bladder cancer research.

**Supplementary Information:**

The online version contains supplementary material available at 10.1186/s12935-021-02422-9.

## Background

Prostate cancer in dogs can be classified into two histopathological groups: prostate adenocarcinoma (PAC) and transitional cell carcinoma (TCC) [1]. Both are typically characterized by metastasis, local invasiveness, androgen-independence and chemoresistance [2, 3], which is why they have been proposed as models for human metastatic castration-resistant prostate cancer (MCRPC) and invasive bladder cancer [4, 5]. Unlike in men, canine prostate cancer is rarely diagnosed [6, 7], making tumor cell lines an indispensable in vitro model system and a vital tool in preclinical research [8, 9]. Notwithstanding their utility, results generated with cell lines are not unconditionally transferable to in vivo conditions. Indeed, through selection by multiple passaging, cell lines adapt to culturing conditions, resulting in differences in gene copy numbers, gene expression and protein synthesis between the cell lines and their original tumor tissues [10–13]. Thus, the suitability of a given cell line as model for a specific feature should be verified before initiating a clinical study. Applications for cell lines as in vitro models are versatile, including research on metastasis, targeted therapy and chemoresistance.

Metastasis involves a complex series of events. Among them, the epithelial-to-mesenchymal transition (EMT) plays a critical role in canine prostate cancer [14], since it promotes invasive growth and enables cell mobility and migration [15]. EMT is also associated with enhanced chemoresistance [16]. Evidently, to be of predictive value, the extent of EMT in cell lines should be comparable to that of the original tumor.

Chemotherapy can follow tumor type or patient-specific approaches [17–19]. Patient-specific therapy aims at targets, which are genes overexpressed by the tumor. Especially receptor tyrosine kinases (RTK) such as those involved in the PI3K-AKT signaling pathway are frequently targeted [20, 21]. Toceranib and masitinib are examples of RTK inhibitors that have been approved for the treatment of mast cell tumors in dogs [18, 20]. A stable expression of the therapeutic target and its downstream molecules in the utilized cell line is essential for conducting a preclinical study [22–24]. Consequently, genomic and transcriptomic profiling of cell lines in direct comparison to their respective original tumor tissues, as performed in the present study, are highly recommended [11, 13]. Tumor type-specific chemotherapy, on the other hand, relies on agents which are effective in the majority of cases in a heterogenous population of patients. Taking into account the enormous intra- and intertumoral heterogeneity of canine prostate cancer in dogs and men [1, 25, 26], chemosensitivity tests conducted on a panel of cell lines are of more practical use than those involving single cell lines [27]. Recognizing this, the United States National Cancer Institute launched a screen of a panel of 60 human tumor cell lines more than 20 years ago; the screen is known as NCI-60 and is still active [27, 28]. For canine TCC, Dhawan et al. and Rathore et al. established a panel of well-characterized K9TCC cell lines [11, 29, 30]. Furthermore, a number of canine PAC cell lines have been profiled for different features as well [2, 31–37].

**Fig. 1:**
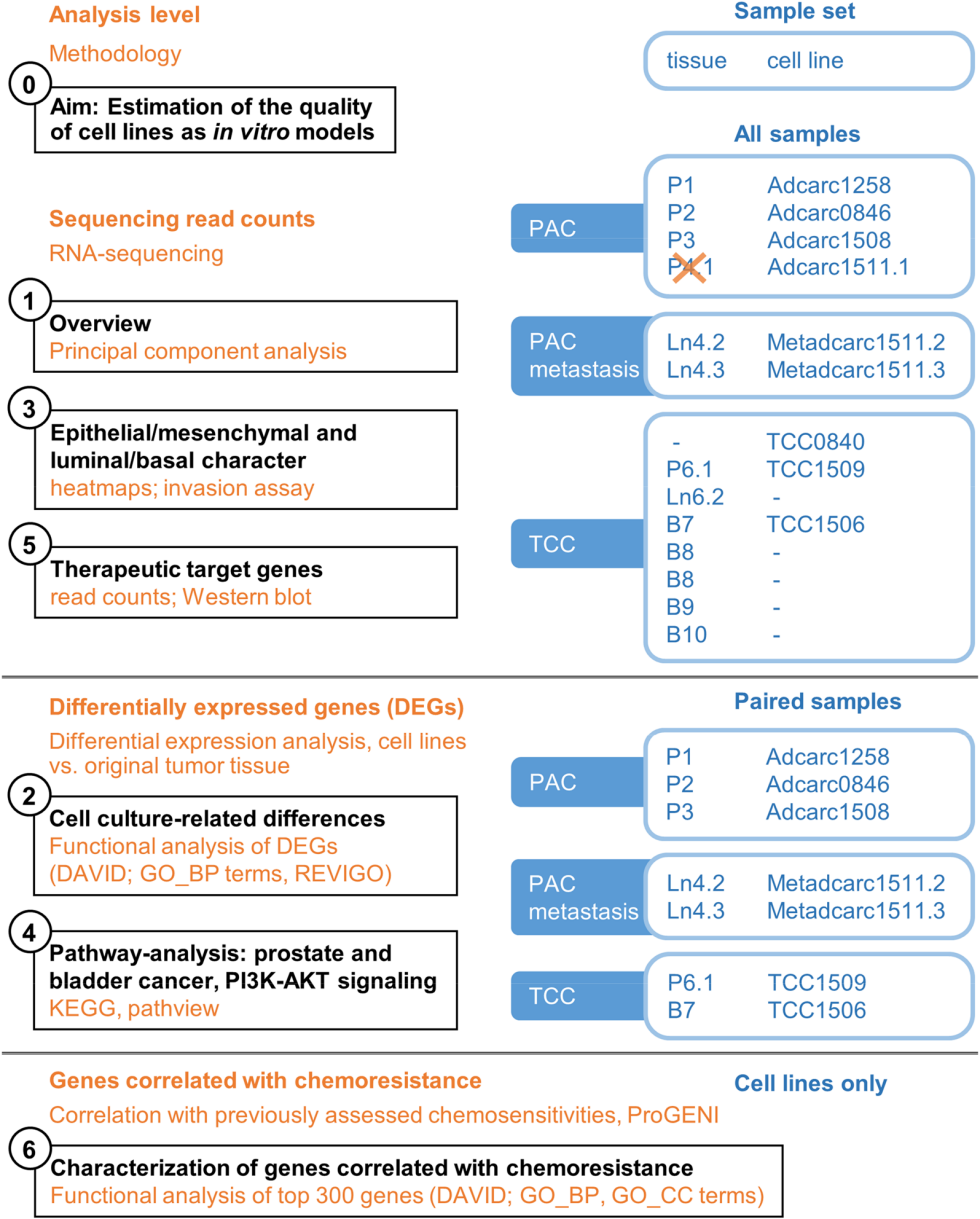
Study design

The present study aims to contribute to the existing knowledge of prostate cancer with the comprehensive transcriptomic characterization of a panel of four PAC, two PAC metastasis and three TCC cell lines and their original tumor tissues. Basic features of this sample set have been reported previously, including medical patient data, histopathology, doubling time, growth behavior, immunophenotype and chemosensitivity towards doxorubicin, carboplatin and meloxicam [37]. Continuing from that point, the cell lines are herein investigated in direct comparison to their original tumor tissue using whole-transcriptome sequencing (Fig. 1). Whole-transcriptome sequencing is a high-throughput technology that is already used in veterinary and comparative oncology [38, 39]. It provides an overview of the complete gene expression landscape and enables detailed single gene expression analyses of therapeutically relevant targets [10, 21, 40, 41]. Specifically, the comparative characterization of the transcriptome of cell lines and their original tumor tissues as performed here has been proved a powerful approach to identify similarities and discrepancies between them [42]. Alas, for many established cell lines, the original tumor tissue is not available or its quality is unsuitable for RNA sequencing and therefore, transcriptome level comparisons are rare [42]. Comparing cell lines to the original tumor tissue in a whole-transcriptome sequencing approach, this study revealed conserved patient-specific characteristics, EMT-properties and downstream PI3K-AKT signaling, along with unique therapeutic target signatures and cell-culture-related adaptations. Together, these data provide suggestions as to which cell line might be the most suitable in vitro model to assess a specific therapeutic approach targeting canine metastatic prostate cancer.

## Materials and methods

### Ethical statement, tissue samples and cell lines

Tissue samples and one fine needle-aspiration biopsy of suspected prostate or bladder carcinomas were collected from ten patient dogs at the Small Animal Clinic, University of Veterinary Medicine Hannover, Foundation, Hannover, Germany between 2003 and 2015 with the consent of the patients’ owners (Table 1). From one PAC patient (P4) and one TCC patient (P6), additional tissue samples of lymph node metastases were collected.

**Table 1:** Patients, tissue samples and cell lines

Patient	Original tissue	Histopathological classification	Cell line	Former name
1	P1	PAC	TihoDPro**Adcarc1258**	CT1258
2	P2	PAC	TihoDPro**Adcarc0846**	DT08/46
3	P3	PAC	TihoDPro**Adcarc1508**	DT15/08
4	P4.1^a^	PAC	TihoDPro**Adcarc1511.1**	none
	Ln4.2	PAC metastasis	TihoDPro**Metadcarc1511.2**	none
	Ln4.3	PAC metastasis	TihoDPro**Metadcarc1511.3**	none
5	n.a	TCC^b^	TihoDProCarc/**TCC0840**	DT08/40
6	P6.1	TCC	TihoDPro**TCC1509**	DT15/09
	Ln6.2	TCC metastasis	None	
7	B7	TCC	TihoDUrt**TCC1506**	DT15/06
8	B8	TCC	None	
9	B9	TCC	None	
10	B10	TCC	None	

Original tissue (P, Prostate; B, urinary bladder; Ln, lymph node); cell lines’ names are explained as institution (Tiho, University of Veterinary Medicine Hannover); species (D, dog); tissue origin (Pro, prostate; Urt, urinary tract (urinary bladder)); diagnosis (Adcarc, adenocarcinoma; Carc, carcinoma; Metadcarc, metastasis of an adenocarcinoma); abbreviations of cell lines written in bold; none, cell lines have not been published yet; n.a., cell line was established from leftover cells from diagnostic fine needle aspiration biopsy, as the patient owners declined surgery and necropsy; ^a^RNA quality not sufficient; ^b^diagnosis by cytology of cells obtained by fine needle aspiration biopsy; former name: cell lines were previously published under different names [43, 44]

Each tissue sample was subsequently divided into three parts: The first part (“original tissue”) was fresh frozen in liquid nitrogen and stored at −80 °C until RNA isolation. The second part was histopathologically classified into PAC or TCC by a certified and experienced pathologist. Modified Gleason scores were between 8 and 10; metastasis confirmed in nearly all cases [37]. Cell lines were established from the third part from four PAC patients, two additional lymph node metastases of a PAC patient (P4), and three TCC tissues [37]. Cell lines were established as previously described (Table 1) [37]. Culturing conditions were Medium 199 (Life Technologies GmbH, Darmstadt, Germany) containing 10% fetal calf serum (FBS Superior, Biochrom GmbH, Berlin, Germany), 200 IU/ml penicillin and 200 mg/ml streptomycin (Biochrom GmbH) at 37 °C in humidified air.

Each cell line was seeded in T25 flasks in triplicate beyond passage 60. At 70–80% confluency, cells were detached with TrypLE™Express (Life Technologies GmbH, Darmstadt, Germany), pelleted, fresh frozen in liquid nitrogen and stored at −80 °C until further use.

In accordance with German national legislation, this study does not require ethical approval, as all tissue samples were collected from: (1) a dog that was euthanized due to poor prognosis; or (2) a bladder cancer that was surgically removed as part of treatment; or (3) leftover material from a diagnostic fine needle aspiration biopsy.

### Isolation of total RNA, library preparation and sequencing

RNA from cell pellets was isolated using the RNeasy Mini Kit (Qiagen GmbH, Hilden, Germany), in accordance with the manufacturer’s protocols. For tissue samples, the AllPrep DNA/RNA/miRNA Universal Kit (Qiagen GmbH) was utilized as previously described [45]. RNA was quantified photometrically on a take3 plate in a Synergy2 plate reader (BioTek Instruments GmbH, Bad Friedrichshall, Germany). Samples with RNA integrity numbers ≥ 5.2 measured with RNA 6000 Nano LabChip on an Agilent Bioanalyzer 2100 (Agilent Technologies Inc., Santa Clara, CA, USA) were further processed for library preparation using the NEBNext Ultra RNA preparation kit (New England Biolabs Inc., Ipswich, MA, USA). Tissue sample P4.1 was excluded due to low RNA integrity numbers in three independent trials of RNA-isolation. Single read sequencing was conducted on an Illumina NextSeq500 platform (Illumina Inc., San Diego, CA, USA) with a read length of 75 bp. For cell line Adcarc1258 and tissue samples P1 and P3, sequencing data of previously published experiments were used (Gene Expression Omnibus database accession identifier [45] GSE122916, samples PT-1 for P1 and PT-6 for P3). For cell line Adcarc1258, sequencing data of triplicates that served as solvent control (0.15% V/V DMSO) were used from a previous study (GSE162832) [46].

### RNA-seq data preprocessing

Sequencing reads were trimmed and filtered using trimmomatic (v0.36, [47]) with parameters “-phred33, HEADCROP:11 LEADING:20 TRAILING:20 AVGQUAL:20 MINLEN:25”. Reads were mapped to the dog reference genome (Ensembl CanFam 3.1) [48] and corresponding gene model annotation (v94) using STAR (v2.5.3, [49]) with parameters: “–sjdbOverhang 100 –outSAMtype BAM SortedByCoordinate –quantMode TranscriptomeSAM GeneCounts”. RSEM (v.1.3.0, [50]) was used to quantify gene expression with parameters “–bam –no-bam-output”. Finally, for each library, read counts derived from multiple lanes were added together.

### Differential expression analysis

Differential expression analysis was conducted with the DESeq2 R package [51]. Only protein-coding genes were considered for the analysis. Read counts from technical triplicates were pooled before the analysis. Differential expression analysis was performed separately for each histopathological classification (PAC, PAC metastasis and TCC), using only samples for which both the original tissue and a cell line were available (Fig. 1). Expression was compared between original tissues and cell lines. The PAC and TCC samples originated from three (P1, P2 and P3) and two (P6 and P7) patients, respectively. The design formula used for the corresponding differential expression analyses controlled for patient effects (“design =  ~ Patient + Type”, where “Patient” stands for the patient identifier and “Type” for the sample type, i.e., “original tissue” or “cell line”). The two PAC metastasis samples were derived from the same patient (P4); in this case, the design formula controlled for the biological replicate. Genes that (i) had an adjusted P-value smaller than or equal to 0.01; (ii) exhibited a fold-change greater than or equal to 2; and (iii) featured ten or more read counts in all original tissue samples or in all cell lines considered in the specific differential expression analysis were regarded as differentially expressed gene (DEG).

### Expression values

Expression values were obtained by applying the regularized logarithm transformation implemented in the rlog() function of the DESeq2 R package [51] to the raw read counts; no experimental design (“design =  ~ 1”) was used for this purpose.

### Visualization of gene expression profiles

Gene expression profiles (z-scores of the regularized-logarithm transformation) were visualized as heatmaps with the pheatmap() function from the homonymous R package [52]. Clustering of genes was performed based on the Euclidean distance, using the complete linkage algorithm.

### Examination of the specific invasive potential of each cell line

An invasion assay was performed to study EMT and metastatic potential of the nine cell lines. Thereby, cells which were allowed to migrate through an artificial basement membrane, attracted by a serum-containing medium, were quantified. In preparation, cell culture inserts with 8 µm pores in transparent PET membranes (Falcon ®, Corning Inc., Corning, NY, USA) were placed into 12 well plates and coated over night with 200 µg/ml basement membrane extract (Cultrex ®, Bio-Techne Corp., Minneapolis, MN, USA) in serum-free Medium 199 (Life Technologies GmbH). Additionally, the cells were starved overnight in serum-free medium. The next day, the starved cells were seeded with a density of 2.5 × 10^5^ cells per insert in serum-free medium. The lower chambers of the wells were filled in duplicates with medium containing 10% serum as attractant, or with serum-free medium as controls. After 48 h of incubation, non-invasive cells were removed from the upper chamber with moistured cotton swabs and two washing steps with PBS. The remaining invasive cells were fixed in 10% formalin for 10 min and permeabilized by methanol for 20 min, both followed by washing twice in PBS. Subsequently, the cells were stained for 2 min with 1% crystal violet, washed three times and allowed to dry. Pictures of three representative areas on each membrane were taken with a DMI600 B microscope (Leica Microsystems, Wetzlar, Germany) in 100× magnification using the LAS AF 2.6.0 software. Finally, the area covered by invasive cells was quantified using Image J 2.0.0. The experiment was conducted three times independently. Means were compared by univariate ANOVA followed by a Ryan-Einot-Gabriel-Welsch post-hoc test using SAS Enterprise Guide® 7.15.

### Functional analysis

Functional analysis of DEGs was performed with DAVID (Database for annotation, visualization and integrated discovery tool) [53, 54], based on Gene Ontology Biological Processes (“GOTERM_BP_DIRECT”), Gene Ontology Cellular Component (“GOTERM_CC_DIRECT”) and a false discovery rate (FDR) of less than 5% as level of significance. The set of all 19,574 protein-coding genes in the dog genome was used as background. Retrieved enriched biological processes (GO_BP terms) were further categorized using REVIGO (reduce + visualize gene ontology) [55].

### Expressed genes

For PAC, PAC metastasis and TCC, a gene was considered expressed if it showed a minimum of ten reads in each cell line sample and/or each tissue sample.

### Visualization of relevant pathways

The fold-changes of the DEGs in the Kyoto Encyclopedia of Genes and Genomes (KEGG) [56–58] prostate cancer pathway (cfa05215), bladder cancer pathway (cfa05219), and PI3K-Akt signaling pathway (cfa04151) were visualized with the Pathview web tool [59]. The analysis was performed using Ensembl gene IDs. When one box represented multiple genes, the mean log2 foldchange was displayed.

### Examination of specific targets at protein level by Western blot

All nine cell lines were washed three times with ice cold PBS and detached by scraping in RIPA buffer containing cOmpleteTM Mini Protease Inhibitor Cocktail (Roche GmbH, Mannheim, Germany). After cell lysis by ultrasound (Bandelin, Berlin, Germany), protein concentrations were determined by Bradford Assay (Bio-Rad Laboratories GmbH, Feldkirchen, Germany). Next, 30 µg cell lysate per well was separated by electrophoresis in Criterion™ TGX™ gels and blotted onto PVDF membranes using the Trans-Blot Turbo Transfer System (Bio-Rad Laboratories GmbH). The membranes were blocked in Odyssey blocking buffer (LI-COR Biosciences GmbH, Bad Homburg, Germany) diluted 1:3 in PBST for one hour at room temperature. Afterwards, the membranes were incubated with primary antibodies against PI3K-p110α (#4249, 1:1000), PI3K-p85 (#4292, 1:1000), Akt (#9272, 1:1000) and β-Actin (#4970, 1:5000) (all Cell Signaling Inc., Danvers, MA, USA) diluted in 1:5 blocking buffer in PBST at 4 °C overnight. The next day, the membranes were washed again, followed by incubation with fluorescence-labeled secondary antibodies (IRDye 800CW goat anti rabbit, LI-COR Biosciences GmbH) for one hour at room temperature. After three further washing steps, fluorescence signals were detected by a LI-COR Imager with Image Studio Lite software (LI-COR Biosciences GmbH). The Western blot analysis was repeated three times with subsequent passages. Protein lysates of the lymphoma cell lines GL-1 (canine), NALM-6 and SEM (both human) served as positive controls.

### Human orthologs to canine genes

Human orthologs of canine genes were identified using the table of orthologous genes retrieved from Ensembl BioMart [60] on November 28, 2018. Specifically, canine Ensembl Gene IDs were used to obtain human Ensembl Gene IDs. Only genes with an orthology confidence score of 1 were considered orthologs. Among 19,574 Ensembl Gene IDs of canine protein-coding genes, 16,065 had an ortholog; in turn, the orthologs involved a total of 15,436 human Ensembl Gene IDs.

### Identification of genes correlated with chemoresistance

Half maximal inhibitory concentrations of doxorubicin and carboplatin for all nine analyzed cell lines measured in metabolic activity and cell count in a previous study [37] were correlated with log2 transformed read counts of the present dataset. The ProGENI correlation [61] was guided by a network obtained from the STRING database for the species canis lupus [62]. To match the gene expression data with the network information from SRTRING, Ensembl gene IDs were converted to Ensembl protein IDs by the bioDBnet conversion web tool [63] prior to analysis.

Furthermore, the top 300 genes ranked by ProGENI for doxorubicin or carboplatin resistance based on either metabolic activity or cell count were subjected to functional analysis with DAVID [53, 54]. Human orthologs of the identified top 300 genes were used, as up to now, the description of biological processes is described in much more detail for humans than for dogs. The complete set of 15,436 human orthologs of all 19,574 protein-coding genes in the dog genome was used as background. The level of significance was set to a false discovery rate (FDR) of less than 5%.

## Results

### PAC and TCC cell lines conserved characteristics of their original tumor tissue despite large culture-induced gene expression changes

**Fig. 2:**
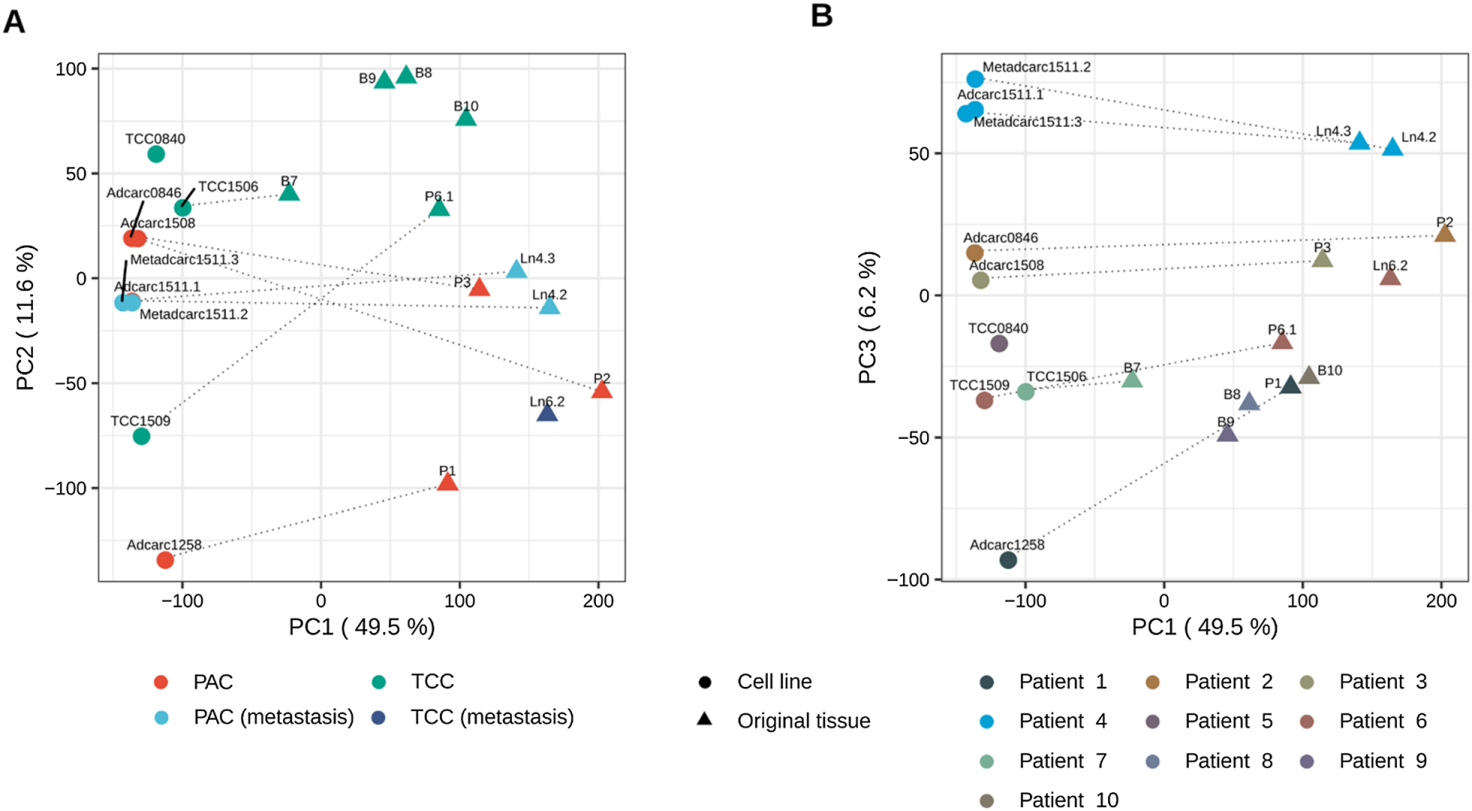
Principal component analysis (PCA) of all samples based on the expression values of 19,574 protein-coding genes. **A** The first principal component (PC1) explaining 49.5% of the variance in the data separates cell lines from tissue samples. **B** PC3 explaining 6.3% of the variance in the data distinguishes between the specific patients. Dashed lines connect the cell line and original tissue of a given patient (“paired samples”)

This study characterized seven cell lines of canine urinary tract cancer in direct comparison to their original tumor tissues. Previous histopathological examination [37] of the same samples had classified three of the tumor samples as PAC, two as PAC metastasis and two as TCC (Table 1). Principal component analysis (PCA) of the transcriptomic profiles of all samples clearly distinguished between cell lines and tissue samples (Fig. 2). Specifically, principal component (PC) 1 explained 49.5% of the variance in the data and separated cell lines from tissue samples; PC2 explained 11.63% of the variance in the data and mainly differentiated between two groups of original tissue samples: PAC, including PAC metastasis, and TCC (Fig. 2A). Based on PC2, the cell lines Adcarc1258 and TCC1509 were relatively far apart from other cell lines corresponding to the same histopathological classification. PC3 explained 6.25% of the variance in the data (Fig. 2B). PC3 distinguished between different patients. Note that for completeness, this analysis also included two cell lines without available tissues (one PAC and one TCC) and four tissue samples from which no cell line had been derived (three TCC and one TCC metastasis).

### Immune response, cell adhesion and angiogenesis were commonly downregulated in PAC and TCC cell lines

**Fig. 3:**
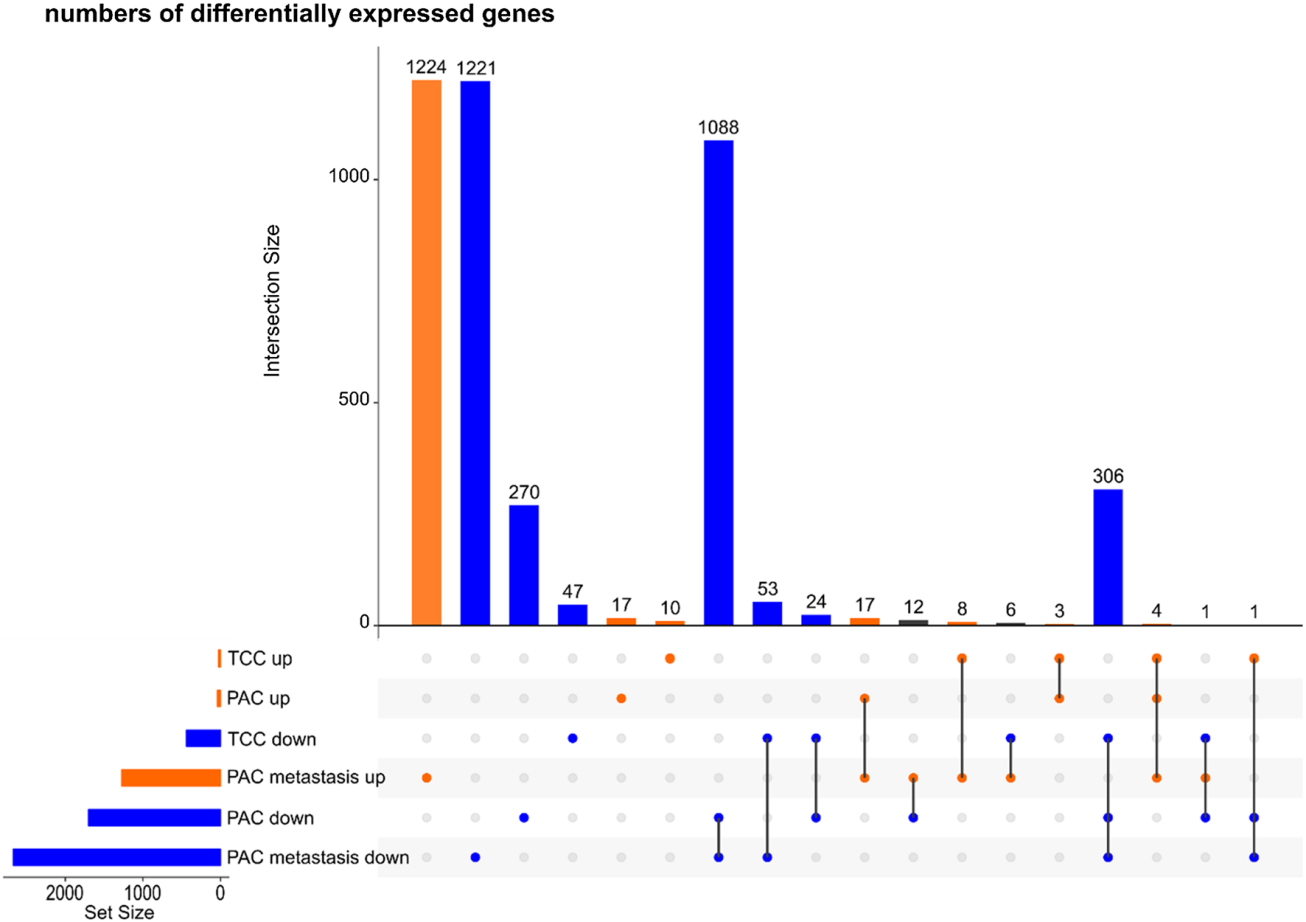
UpSet [64] plot showing the numbers of up- and downregulated genes for each histopathological classification. Intersections are represented as disjoint sets

Separate analyses for PAC, PAC metastasis, and TCC revealed 1743, 3941 and 463 DEGs, respectively, between the cell lines and their original tumor tissues when controlling for patient-specific effects (Methods). Independently of the histological classification of the samples, biological processes (GO_BP terms) enriched among the identified DEGs were associated with immune response, cell adhesion and angiogenesis/endodermal cell differentiation (Additional file 1). In addition, the DEGs in PAC samples were enriched in genes related to collagen fibril organization, calcium ion transport, lipoprotein metabolism and negative regulation of viral genome replication. Most of the DEGs (98%, 68% and 94%, respectively) were downregulated in the cell lines relative to the original tissue samples (Fig. 3). The majority of DEGs (2445; 62%) in the PAC metastasis samples were not differentially expressed in the PAC or TCC samples. In contrast, only 287 (17%) and 57 (12%) of the DEGs in the PAC and TCC samples were exclusive to PAC and TCC, respectively. A total of 310 DEGs were shared among all three histopathological classifications. The majority of DEGs, specifically 1300 (75%) in PAC, 1599 (41%) in PAC metastasis and 344 (74%) in TCC, featured large log_2_ fold-changes, greater than 5 or smaller than −5.

### Cell lines preserved the epithelial or mesenchymal character of their original tumors

**Fig. 4:**
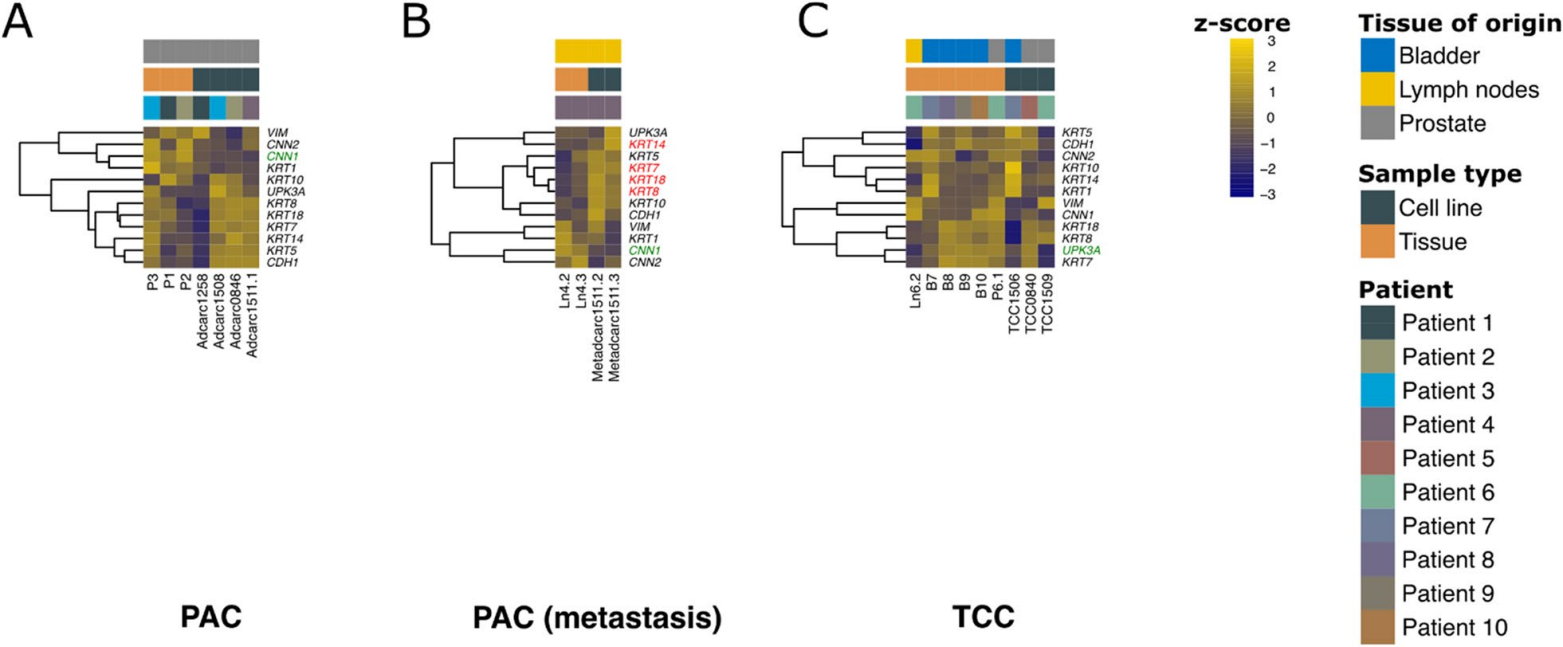
Expression of genes previously analyzed by immunohistochemistry or immunocytochemistry [37] in PAC (**A**), PAC metastasis (**B**) and TCC samples (**C**). DEGs are indicated in green (downregulated in the cell lines relative to their original tissues) and red (upregulated)

To validate the gene expression data and assess if the cell lines conserve the epithelial or mesenchymal character of their original tissue, an analysis of thirteen genes that had been previously investigated by immunohistochemistry or immunocytochemistry [37] was carried out. With the exception of PAC metastasis (Fig. 4B), most of these genes were not differentially expressed. In the PAC metastasis cell lines, the cytokeratins 7, 8, 14 and 18 were upregulated, while *CNN1* was downregulated (Fig. 4B). In contrast, in the PAC group (Fig. 4A), cell line Adcarc1258, its tumor tissue P1 and the tumor P2 were noticeable due to their relatively low expression of the epithelial cytokeratin genes, *CDH1* and *UPK3A*. Additionally, Adcarc1258 showed a relatively high expression of the mesenchymal marker *VIM* in trend, while the other PAC cell lines and tissue P3 behaved in the opposite manner. Similarly to Adcarc1258, the TCC cell line TCC1509 and the corresponding lymph node tissue Ln6.2 of the same patient had a remarkably high expression of the mesenchymal marker *VIM* (Fig. 4C). In general, TCC1509 appeared to be more similar to the metastasis tissue Ln6.2 than to its original tumor tissue (P6.1, Fig. 4C). Another remarkable observation among TCC samples was the fact that TCC1506 showed a relatively low expression of cytokeratins 8 and 18 (Fig. 4C).

### While TCC cell lines conserved basal and luminal characteristics of the original tumor, both were downregulated in PAC metastasis cell lines, and basal features were downregulated in PAC cell lines

**Fig. 5:**
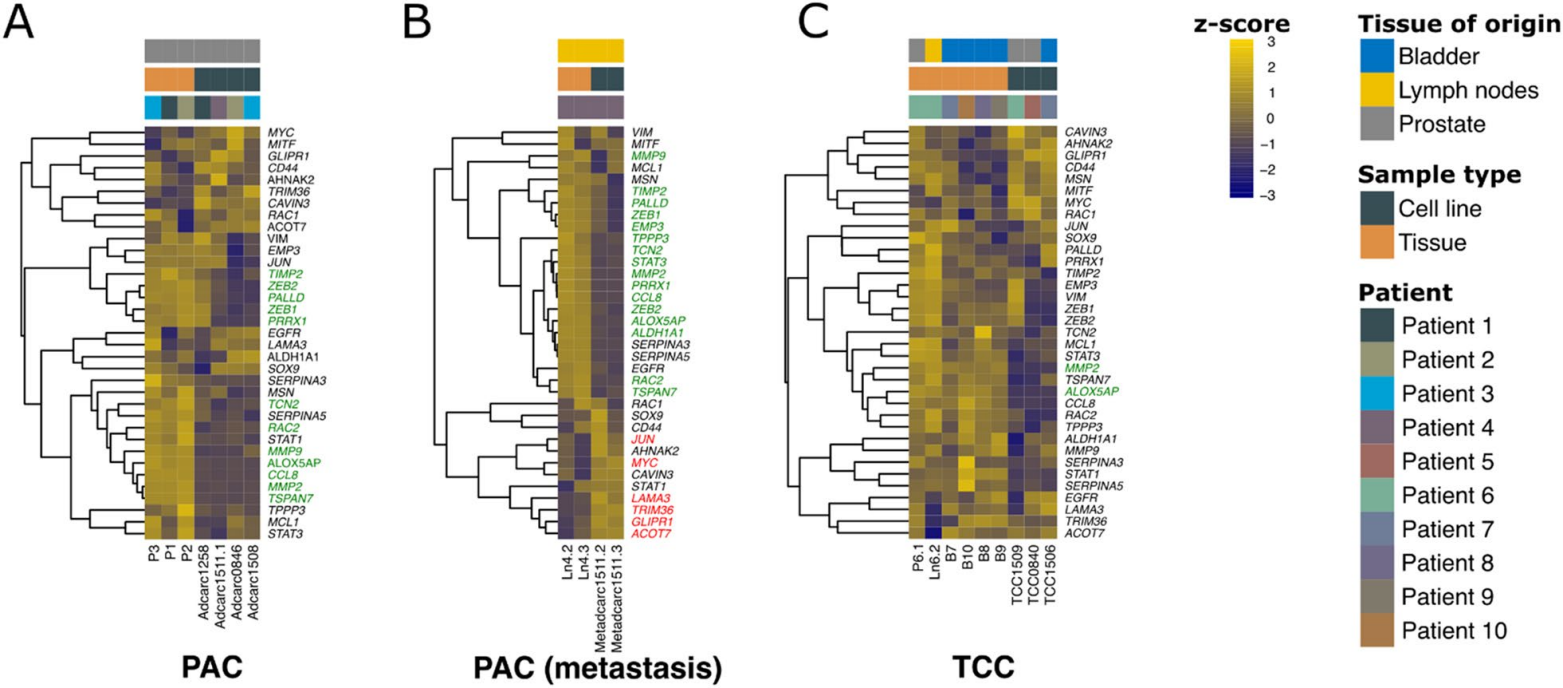
Expression of selected set of basal cell markers [65] in PAC (**A**), PAC metastasis (**B**) and TCC samples (**C**). DEGs are indicated in green (downregulated in the cell lines relative to their original tissues) and red (upregulated)

In a previous analysis, Dhawan et al. were able to classify their canine TCC tissue samples into a luminal and a basal subtype based on their gene expression profile [65]. Thus, they compiled two gene lists characteristic for basal and luminal subtypes of canine and human bladder cancer [65]. Similarly, human prostate cancer can be segregated into luminal and basal [66]. When analyzing the list of 35 genes associated with basal subtypes in the dataset of the present study, 11, 22 and two genes were differentially expressed in PAC, PAC metastasis and TCC, respectively (Fig. 5). Cell lines and tissue samples generally showed different gene expression profiles (Fig. 5).

**Fig. 6:**
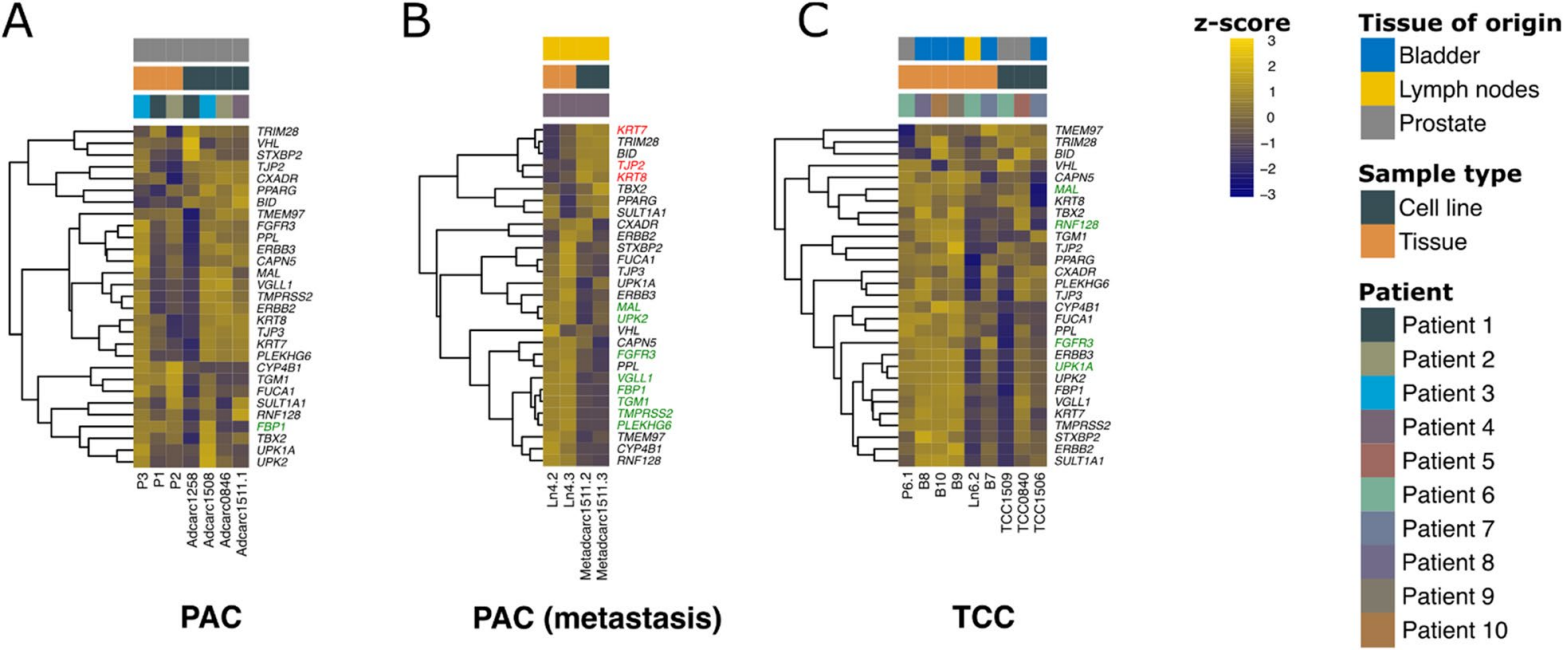
Expression of a selected set of luminal cell markers [65] in PAC (**A**), PAC metastasis (**B**) and TCC samples (**C**). DEGs are indicated in green (downregulated in the cell lines relative to their original tissues) and red (upregulated)

With the exception of the PAC metastasis samples (Fig. 6B), most of the 29 genes associated with luminal subtypes of canine and human bladder cancer [65] were not differentially expressed. Regarding the extent of luminal characteristics, the individual patients differed. For example, the PAC cell line Adcarc1258 and its corresponding tumor tissue P1 showed generally low expression for these genes (Figs. 5 and 6). The same phenomenon was observed in the TCC group for cell line TCC1509 and the respective lymph node metastasis tissue Ln6.2 (Fig. 6C). Consistently, compared to the respective tissues, both PAC metastasis cell lines showed a less luminal character (Fig. 6B).

### Cell lines with low epithelial and luminal marker gene expression were highly invasive

**Fig. 7:**
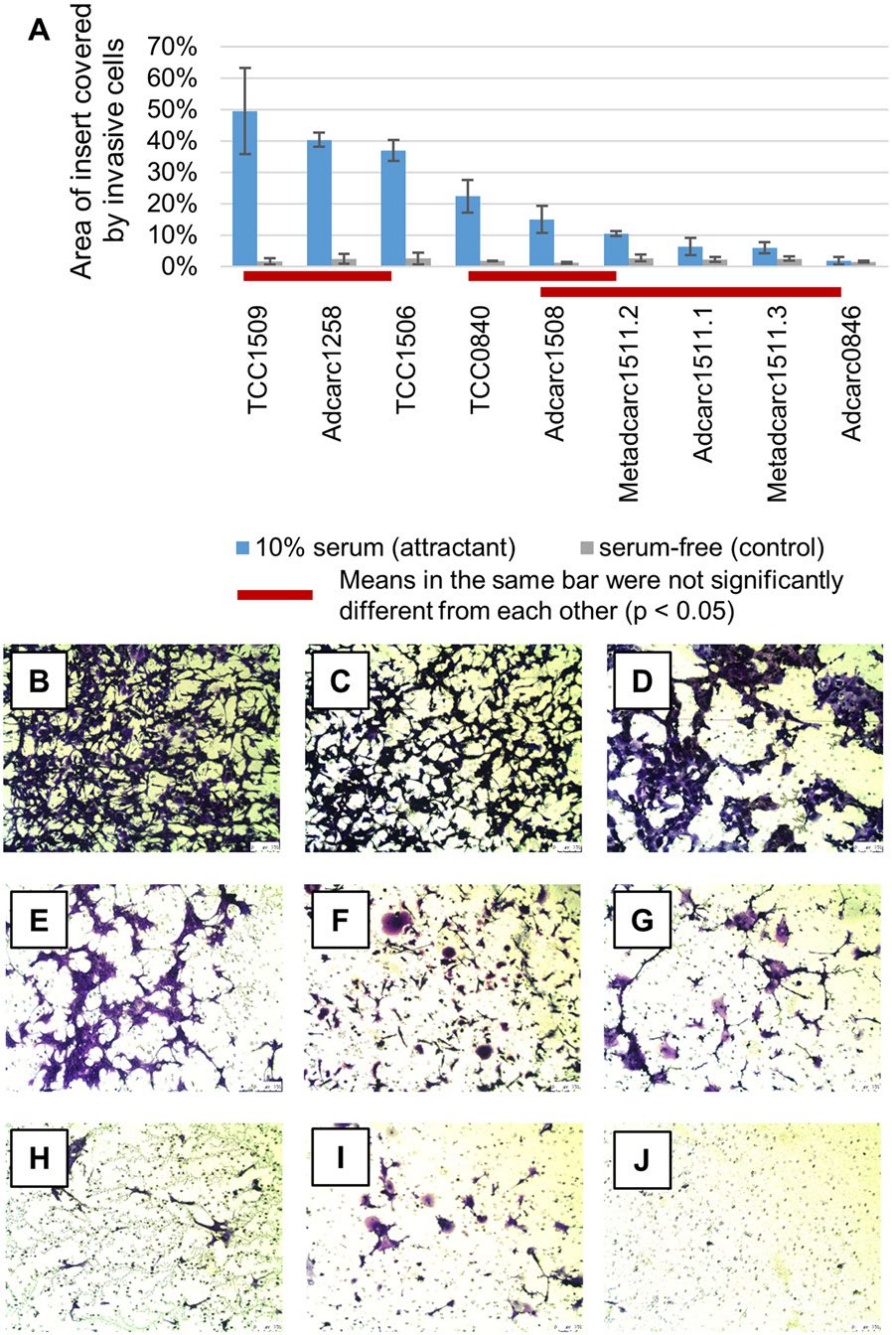
Invasive potential of analyzed cell lines determined based on the number of serum-starved cells that invaded a second medium compartment containing 10% serum through an artificial basement membrane. **A** Blue bars depict the percentage of membrane area covered by invasive cells. Gray bars show negative controls with serum-free medium in the second compartment. ANOVA with post-hoc test assigned the cell lines to groups (red bars). Means within one group were not significantly different from each other (p < 0.05). Cell lines belonging to different groups showed statistically significant differences. **B**–**J** Representative images of invaded cells on the membranes, 100× magnification, scale bars represent 250 µm, B TCC1509, C Adcarc1258, D TCC1506, E TCC0840, F Adcarc1508, G Metadcarc1511.2, H Adcarc1511.1, I Metadcarc1511.3, J Adcarc0846

An invasion assay was conducted to verify the mesenchymal transition suggested by the transcriptomic data. Invasive potential was measured as the area of the artificial basement membrane covered by invasive cells (Materials and Methods). Consistent with their high expression of the mesenchymal marker *VIM* and low expression of epithelial and luminal markers (Figs. 4 and 6), TCC1509 and Adcarc1258 exhibited the highest invasive potential among all analyzed cell lines (Fig. 7). Also TCC1506, which was notable for its low expression of cytokeratins 8 and 18, exhibited a highly invasive character. However, the total number of invaded cells in TCC1509 and Adcarc1258 is likely to be higher than in TCC1506 due to their relatively small cell size (Fig. 7B–D). The metastasis-derived cell lines, in which epithelial cytokeratin genes were upregulated (Fig. 4), were among the least invasive cell lines.

### Cell lines conserved prostate and bladder cancer pathway gene expression, while certain prostate-specific genes were not expressed or downregulated

**Fig. 8:**
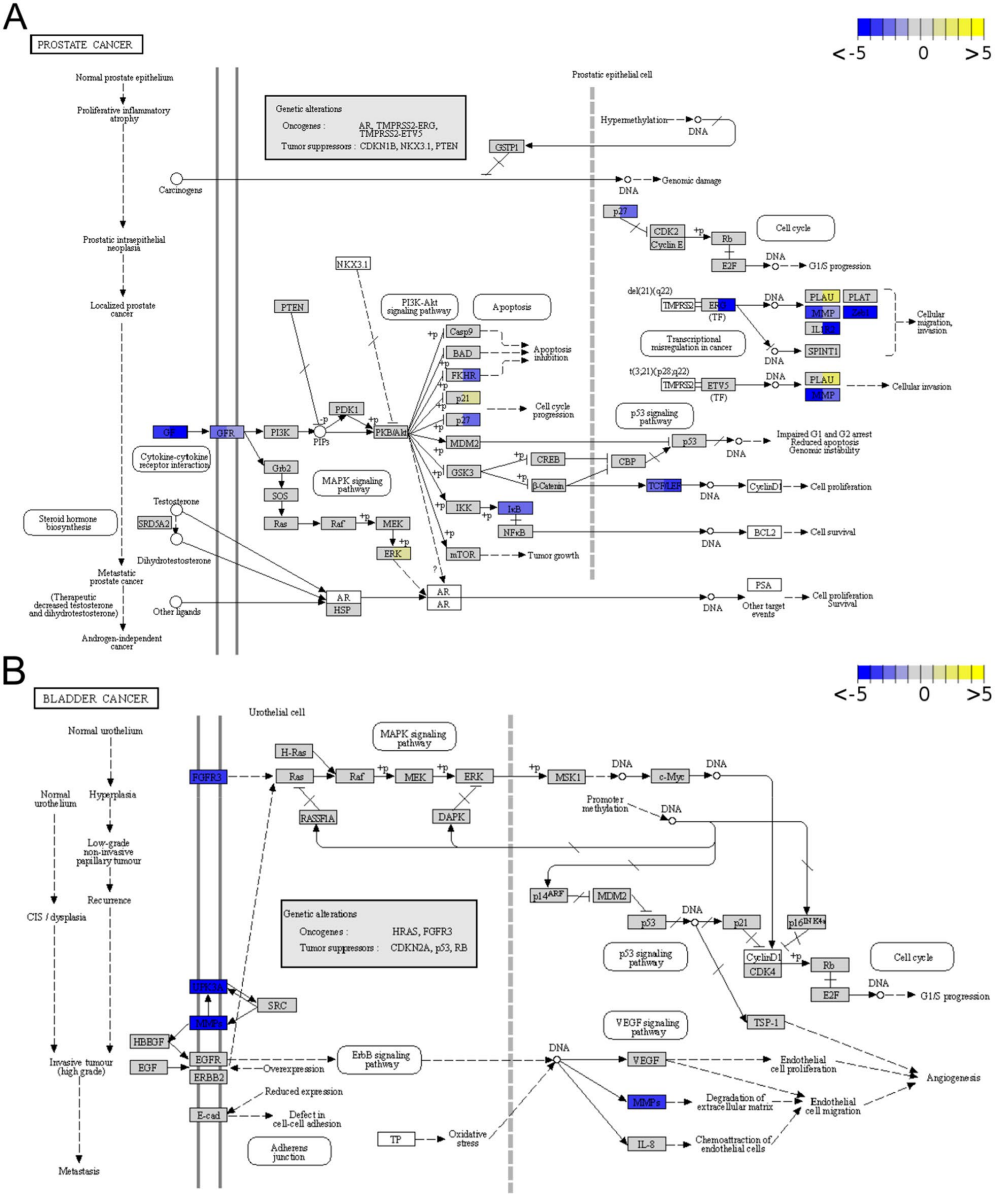
The KEGG prostate cancer pathway hsa05215 (**A**) and bladder cancer pathway hsa05219 (**B**) [67]. The boxes represent genes. The colors visualize log_2_ fold-changes of genes differentially expressed in cell lines relative to tissue samples, with yellow standing for upregulation and blue for downregulation. Boxes representing multiple genes display the mean log_2_ fold-change. The visualization of deregulation was capped at log_2_ fold-changes of ± 5. Boxes in the prostate cancer pathway (**A**) are divided into PAC on the left and PAC metastasis, while log_2_ fold-changes in the TCC cell lines are shown in the bladder cancer pathway (**B**). With the exception of *TMPRSS2*, *CCND1* and *BCL2,* whose Ensembl identifiers (ENSCAFG00000010190, ENSCAFG00000010700 and ENSCAFG00000000068) were not recognized by the visualization tool, genes depicted in white were not considered expressed

To further verify whether the cell lines reflect the tumor of origin, special focus was laid on the prostate and bladder cancer pathway genes. Of 97 genes listed in the KEGG [56–58] prostate cancer pathway (cfa05215) and 41 in the bladder cancer pathway (cfa05219), 89 and 39, respectively, were expressed at non-negligible levels (Methods) in the overall sample set. Among those, 14 genes in the prostate cancer pathway were differentially expressed in PAC samples and 35 in PAC metastasis samples (Fig. 8A). The differential expression within the prostate cancer pathway in PAC cell lines was mostly focused on *MMP9* and the group of growth factors and their receptors (GFR), also known as RTK. DEGs in the metastasis cell lines were spread over the entire prostate cancer pathway, including the axes of carcinogenesis, RTKs and downstream molecules, and the hormonal axis (Fig. 8A, Table 2). Besides the pathway genes, hormonal receptors and other genes are of diagnostic and prognostic value in human and canine prostate cancer and were therefore of special interest in the present dataset (Table 2). Among these, *ESR1*, *PGR* and *AMACR* were not differentially expressed. In contrast, the androgen receptor, *KLK2*, *NKX3-1* and *ESR2* were not expressed and the prostate-specific markers *ACPP, FOLH1, PSCA* and *GSTP1* were downregulated in the PAC and PAC metastasis cell lines.

**Table 2:** *Expression status* of selected genes with diagnostic or prognostic value in human and canine prostate cancer in the analyzed cell lines compared to original tumor tissues

Gene	Ensembl gene ID	PAC	PAC metastasis	TCC
**Hormonal receptors and genes associated with androgen-independence**				
AR	ENSCAFG00000016656	n.e	n.e	n.e
ESR1	ENSCAFG00000000430	e.	e.	e.
ESR2	ENSCAFG00000015846	n.e	n.e	n.e
PGR	ENSCAFG00000003978	e.	e.	e.
AURKA	ENSCAFG00000029331	e.	e.	e.
HSD3B2	ENSCAFG00000010039	n.e	n.e	n.e
HNF1A	ENSCAFG00000010502	e.	e.	e.
HNF4G	ENSCAFG00000008285	e.	e.	e.
MYCN	ENSCAFG00030012614	n.e	n.e	n.e
SPINK1	ENSCAFG00000006647	n.e	n.e	n.e
**Prostate-specific markers**				
KLK2	ENSCAFG00000002907	n.e	n.e	n.e
NKX3-1	ENSCAFG00000009107	n.e	n.e	n.e
ACPP	ENSCAFG00040021029	Down	Down	e
FOLH1	ENSCAFG00000024888	Down	Down	e
TP63	ENSCAFG00000013961	e.	e.	e.
AMACR	ENSCAFG00000018831	e.	e.	e.
FASN	ENSCAFG00000006006	e.	e.	e.
SLC45A3	ENSCAFG00030008817	e.	e.	e.
PSCA	ENSCAFG00000032433	Down	Down	e.
GSTP1	ENSCAFG00000011349	Down	Down	e.
HOXB13	ENSCAFG00000016870	Up	Up	e.
GOLM1	ENSCAFG00000001350	Up	Up	e.

e., expressed at non-negligible levels, but not differentially expressed; n.e., not expressed; up/down, up- or downregulated in the cell lines of the respective histopathological classification

Only three genes in the bladder cancer pathway were differentially expressed (in particular, they were downregulated) in TCC samples, including the urothelial marker *UPK3A* (Fig. 8B). In general, irrespective of the histopathological classification, the DEGs were not enriched in genes of any of these pathways.

### PAC, PAC metastasis and TCC cell lines conserved the expression of certain therapeutic targets, as well as the PI3K-AKT signaling cascade

**Fig. 9:**
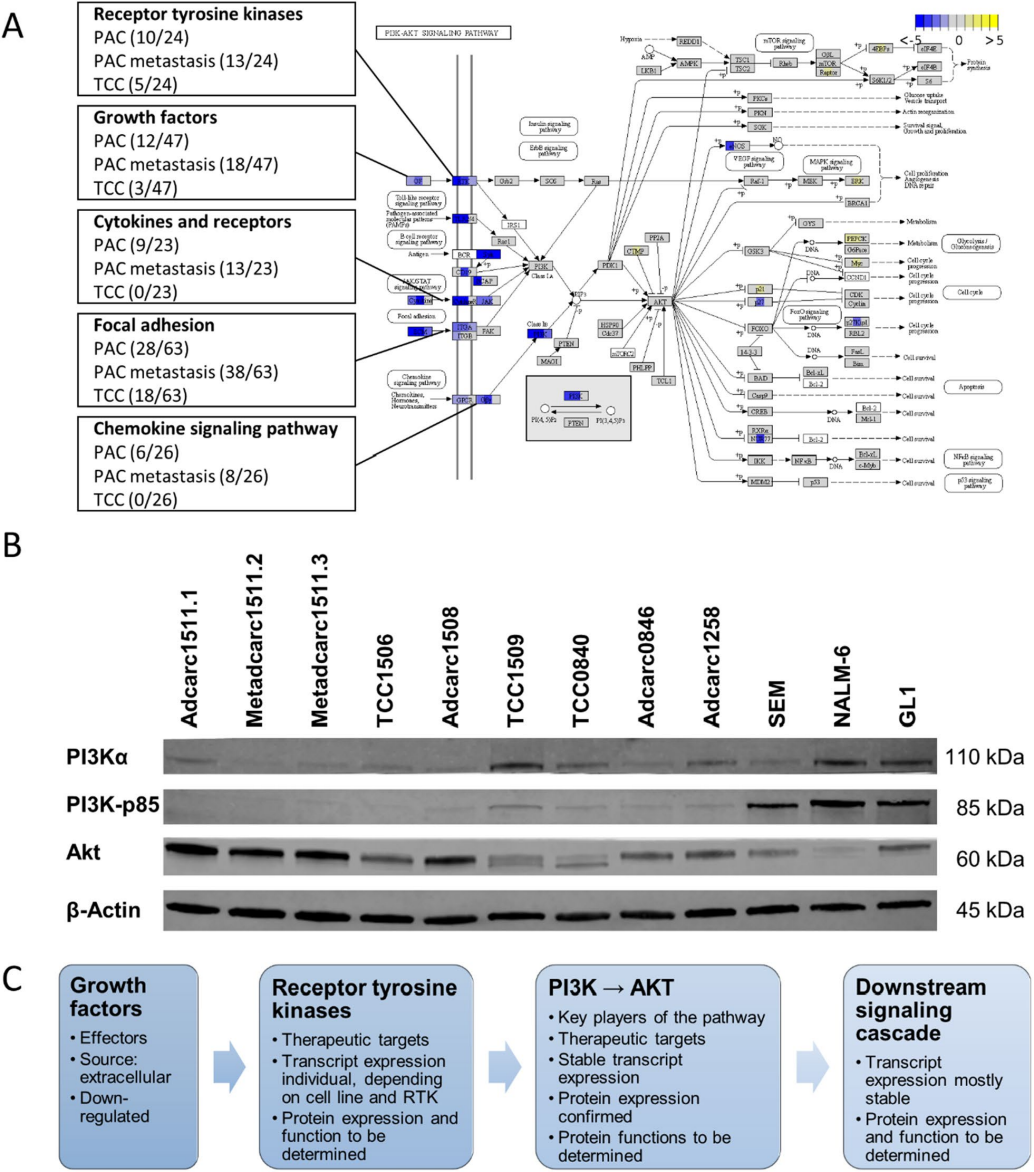
**A** The KEGG PI3K-AKT signaling pathway (cfa04151) [67]. The boxes are divided into three parts depicting DEGs in PAC, PAC metastasis, and TCC cell lines from left to right. With the exception of *BCR*, *IRS1, CCND1* and *BCL2,* whose Ensembl identifiers (ENSCAFG00000013773, ENSCAFG00000010397, ENSCAFG00000010700 and ENSCAFG00000000068) were not recognized by the visualization tool, genes depicted in white were not considered expressed. The boxes on the left summarize the number of identified genes in the growth factors (GF), RTK, cytokines and receptors, focal adhesion and the chemokine signaling pathway which are differentially expressed in each histopathological classification (See Fig. 8 for details). **B** Western blot analysis using antibodies against PI3Kα, PI3K-p85, and Akt. Membranes were cut prior to antibody incubation and cropped to be displayed together. **C** Summary of the findings in the PI3K-AKT signaling pathway

The PI3K-AKT signaling pathway (cfa04151) is an important regulator of the cell cycle and the focus of numerous therapeutic approaches [20, 21, 68]. Consequently, verifying that the genes involved in this pathway are expressed at similarly levels in the cell lines that are being considered as model systems relative to their original tissues is pivotal for preclinical studies. Of the 370 genes in the PI3K-AKT signaling pathway, 77, 124 and 29 were differentially expressed in PAC, PAC metastasis and TCC cell lines, respectively (Fig. 9). Irrespective of the histopathological classification, all sets of DEGs were enriched in genes in this pathway, and like the majority of the DEGs, most of the DEGs in the PI3K-Akt signaling pathway were downregulated.

**Fig. 10:**
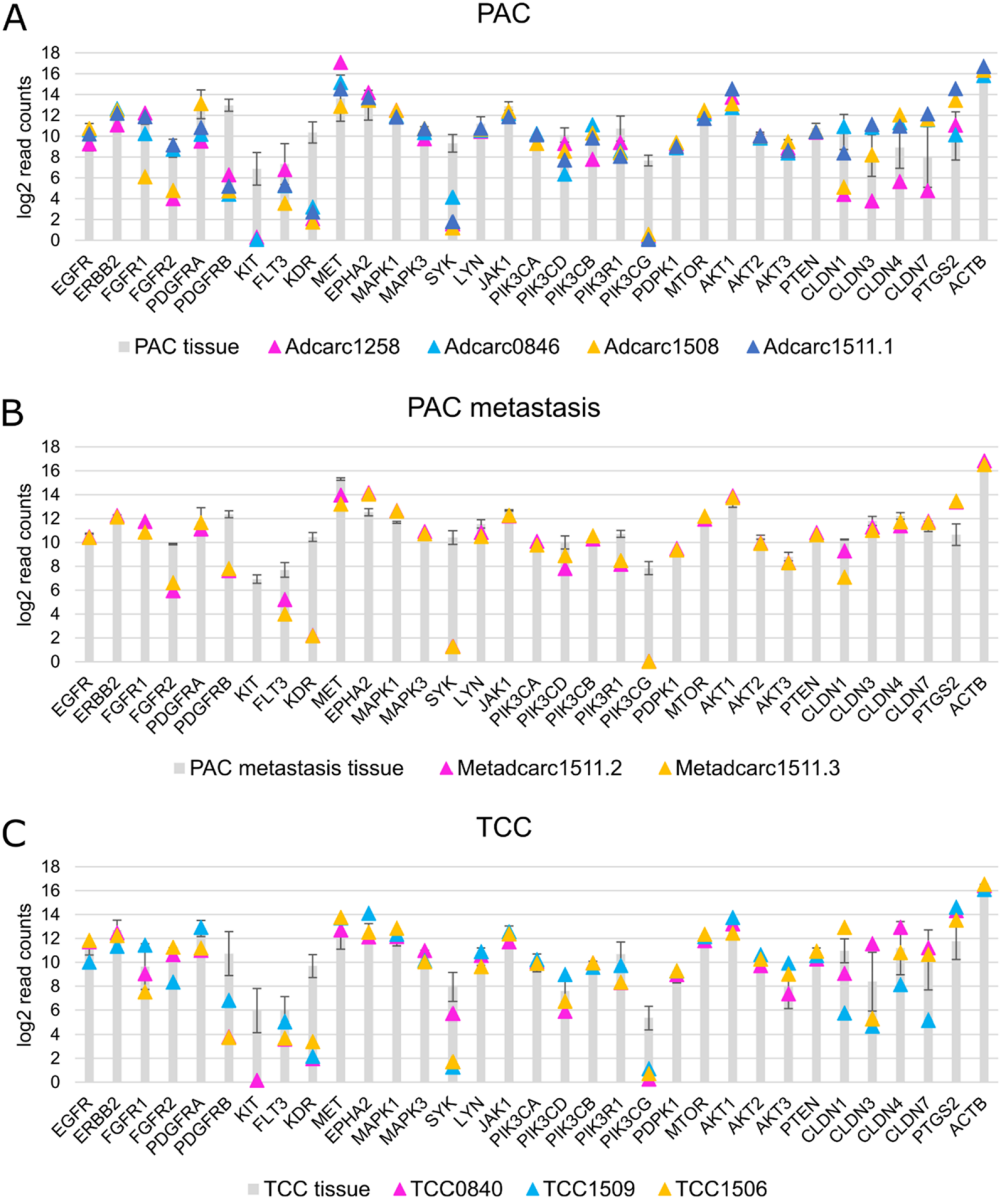
Log_2_ read counts of therapeutically relevant RTK genes, *PI3K* isoforms, and downstream molecules in the PI3K-Akt signaling pathway, as well as claudin genes 1, 3, 4 and 7, and the therapeutic target *PTGS2*. Cell lines were depicted as triangles, compared with averaged log_2_ read counts of all tissues of the same histopathological classification, represented as gray columns

Interestingly, *PI3K* and *AKT*, the key players of the pathway, were not differentially expressed. Moreover, their expression profiles could also be validated at protein level by performing a Western blot (Fig. 9B). Thus, in agreement with the transcriptomic data, AKT produced strong bands, especially in Adcarc1511.1, which featured relatively high transcript levels of *AKT*. Analogously, the weaker bands observed for PI3Kα and PI3K-p85 were in correspondence with their lower transcript levels. In particular, the concordance between mRNA and protein level was the most apparent in TCC1509, which featured the highest expression of *PIK3R1*/PI3K-p85. In addition, only a few genes in the subsequent downstream signaling cascade were differentially expressed, almost exclusively in PAC metastasis. Upstream of *PI3K* and *AKT*, of the 24 RTKs—the activators of the pathway and frequently targeted genes—10, 13, and 5 were differentially expressed in PAC, PAC metastasis and TCC, respectively. Thus, the expression of *KIT*, the main target of Masitinib and Toceranib in canine oncology [18, 20], appeared to be lost in culture. Nevertheless, other RTKs like *ERBB2* and *EGFR* were not differentially expressed. Other targets showed varying gene expression levels in the different cell lines (Fig. 10, Additional file 4). *PTGS2*, which encodes COX-2, was also expressed, and even upregulated in the cell lines.

### Resistance towards doxorubicin was associated with G2/M cell cycle transition, cell membrane biosynthesis and tumor microenvironment, while carboplatin resistance correlated with histone, m- and tRNA processing, TGFB receptor signaling and phagocytosis

Currently, there are no explicit treatment recommendations for canine prostate cancer. Therefore, the chemosensitivity of all nine analyzed cell lines to doxorubicin and carboplatin in metabolic activity and cell counts was assessed in a previous study [37]. In a next step, the chemosensitivity was herein correlated with the gene expression data, guided by known protein networks from the STRING database [62]. The top 300 genes correlated with doxorubicin resistance regarding metabolic activity (Additional files 2 and Additional file 3) were associated with mitosis, especially G2/M cell cycle transition and processes during the M phase, tRNA processing, platelet degranulation and cell membrane biosynthesis. Genes correlating with doxorubicin resistance in cell count were associated with tumor microenvironment, including cell adhesion, extracellular matrix organization, collagen catabolism and the integrin-mediated signaling pathway.

Carboplatin resistance regarding metabolic activity correlated with genes associated with histone deacetylation, glucose transport, oligosaccharide biosynthesis, TGFB receptor signaling pathway, mRNA 3prime-end processing and tRNA splicing. The set of the top 300 genes correlated with carboplatin resistance in cell count was associated with small GTPase mediated signal transduction, which includes numerous member genes of the RAS family of oncogenes. Furthermore, this set of genes was associated with movement of cell or subcellular components, phagocytosis, cytosol and centriole.

Additionally, other well-known multi-resistance genes were examined in the dataset. Of the three genes *ABCB1*, *ABCC1* and *ABCG2*, coding for the multi-drug transporters MDR1, MRP and BCRP, *ABCB1* was upregulated in PAC metastasis and TCC, while *ABCG2* was downregulated in PAC and PAC metastasis.

## Discussion

An important decision in the planning of in vitro studies in prostate and bladder cancer research is the choice of an appropriate model system. Furthermore, only a detailed understanding and characterization of these tumor entities and the applied model systems allow a reasonable interpretation of the obtained results. The optimal reference for characterizing a cell line is the original tissue from which it was initially derived. However, in most cases, such a sample is either not available or not suitable for RNA-seq, and thus, such studies are scarce [42]. The sample set analyzed herein, consisting of canine cell lines derived from four PAC, two PAC metastases and three TCC and their original tumor tissues, provides a rare opportunity.

In general, the present study found that tumor-, subtype- and patient-specific features were conserved in the cell lines compared to their original tissue samples, while genes associated with immune response, cell adhesion and angiogenesis were downregulated. This is consistent with the results by Perry et al., who compared two human squamous cell carcinoma cell lines to their original tumor tissues [42]. Importantly, regarding the tumor entity, only few deviations were found between cell lines and tissues regarding the prostate and bladder cancer pathways.

Similar to other whole-genome transcriptome studies in human PAC [10] and canine lymphoma [69], the current analysis identified a relatively large number of DEGs. In contrast to those studies in which tumor tissues and cell lines originated from different patients, the observations made here cannot be attributed to patient-specific features or different tumor stages. Instead, there are a few possible explanations for the massive down-regulation of genes detected in the present analysis. First, cell lines are cultured in the absence of an immune system and with unlimited access to oxygen and nutrients. Second, cell lines are frequently detached in order to be passaged. Accordingly, processes such as immunoevasion, collagen fibril organization, angiogenesis and cell adhesion become non-essential and are downregulated. Nevertheless, it is worth mentioning that downregulation does not necessarily imply loss of function. Indeed, human tumor cell lines can reactivate genes associated with extracellular matrix organization, angiogenesis and cell adhesion in three-dimensional cultures or in mouse xenografts [70–72]. Similarly, Adcarc1258 is able to form three-dimensional spheroids with a modified gene expression compared to adherent Adcarc1258 cells and induces tumors in immunodeficient mice [73, 74].

The amount of tumor microenvironment and the degree of heterogeneity of the tissue samples may have also contributed to the differences in the number of DEGs observed for each histopathological classification. Thus, while previously published histopathological images of metastasis tissues Ln4.2 and Ln4.3 show lymphoid and connective tissue in addition to the tumor cell population, TCC tissue B7 consists almost exclusively of tumor cells [37]. Consistently, Ln4.2 and Ln4.3 were further apart from their respective cell lines on PC1, which distinguished between cell lines and tissue samples (Fig. 2), than B7 was from its own. A microdissection of the tissue samples [75] prior to RNA isolation and sequencing would help to focus this analysis on the changes the pure tumor cell population underwent in the culturing process. Together with tumor microenvironment, patient heterogeneity may have contributed additionally to the variance in the measured data.

Despite the abovementioned adaptations to culturing conditions, the cell lines preserved the luminal or basal tumor subtypes and the epithelial or mesenchymal characteristics of the tumor. Both findings are consistent with observations from gene expression analyses of human cancer cell lines of various tumor entities [40, 76]. Especially Adcarc1258 and TCC1509 showed a remarkable extent of mesenchymal features regarding the expression pattern of luminal, basal and claudin genes. In this respect, Adcarc1258 displayed similarities to its original tumor tissue and TCC1509 to the respective lymph node metastasis Ln6.2. Consistently, their morphology and growth behavior in cell culture is characterized by high cell mobility, cell protrusions, low adherence, the tendency to form spheroids [37], and highest invasive potential. With the exception of the intermediate cell line TCC1506, the other six cell lines were significantly less invasive and feature a more epithelial, cobblestone-like growth behavior [37]. Chaffer et al. showed a similar relationship between EMT and invasive potential in vitro for human bladder cancer cell lines [77]. However, contrary to the expectation, the cell lines with the highest metastatic potential in in vivo mouse models [77] featured epithelial characteristics in vitro. In order to establish distant colonies, metastatic tumor cells reverse EMT and revert to an epithelial phenotype [78]. This epithelial-mesenchymal plasticity could explain the low invasive potential of the two metastasis cell lines. Although the phenotype and doubling times of both PAC metastasis cell lines Metadcarc1511.2 and Metadcarc1511.3 were completely different from the primary tumor cell line Adcarc1511.1 of the same patient [37], the similarity of all samples from this patient was striking (Fig. 2). In other words, differences in gene expression profiles between PAC tumors of different individuals were much greater than differences between primary tumor and metastasis. These three cell lines offer the rare opportunity to evaluate EMT or perform chemosensitivity tests in cell lines established from primary tumors and metastases of the same patient, preventing patient-specific differences from confounding the results.

The androgen receptor is the most important driver in human prostate cancer. Despite initial success of androgen-deprivation therapy, many men develop androgen-independent MCRPC, in which mutations or splice variants enable the tumor to continue to rely on androgen receptor signaling [79]. Due to its androgen-independence, canine prostate cancer has been proposed as biological model for MCRPC [5]. In contrast to MCRPC, and consistent with other reports [2, 3, 31, 34–36, 39, 80], in the present study the androgen receptor was found to be not expressed in canine prostate cancer tissues and cell lines. The divergent expression of hormonal receptors in human and canine prostate cancer [81–84] argues against the suitability of canine prostate cancer as a model for androgen receptor signaling in human MCRPC. Furthermore, because prostatic TCC is rare in men [1, 82], only canine PAC can be seen as model for MCRPC. Distinction between PAC and TCC, which is also important for therapeutic decisions [1], can be achieved on the basis of the expression of the urothelial marker *UPK3A* [85]. Interestingly, the current analysis observed that *UPK3A* was downregulated in the analyzed TCC cell lines. Furthermore, and in contrast to other canine TCC cell lines [86], the UPK3A protein was not detectable by immunohistochemistry using an antibody with confirmed species immunoreactivity in any of the analyzed samples [37]. A possible reason for this is the loss of UPK3A immunoreactivity in high grade TCC [87].

Although cell lines are common model systems for targeted chemotherapy, results obtained with cell lines are not always reproducible in clinical trials [24]. The main explanation for this is that cell lines undergo clonal selection and therefore often no longer reflect the tumor’s specific target expression [24]. The PI3K signaling pathway is of particular interest in canine prostate cancer [39] and chemotherapy in general [20, 21]. As an important result, the key players of the PI3K signaling pathway, *PI3K* and *AKT*, were not differentially expressed between cell lines and tissues (except for *AKT1* and *PIK3CD* in PAC metastasis, log_2_ fold-changes 1.0 and −1.9). Furthermore, only few downstream genes were differentially expressed, almost exclusively in the PAC metastasis cell lines. Although the functionality of the corresponding proteins warrants further investigation, these findings indicate a good model suitability of the analyzed cell lines for the downstream *PI3K* signaling cascade. This observation is surprising given that numerous RTKs—direct activators of *PI3K*—were downregulated. In general, each cell line exhibited its own individual therapeutic marker signature. For instance, the RTK *FGFR2* is targeted by erdafitinib, an inhibitor for the treatment of human metastatic bladder cancer [88]. Although *FGFR2* was generally downregulated in PAC cell lines, Adcarc0846 and Adcarc1511.1 appear to have similar *FGFR2* expression levels relative to their original tissues. Thus, those two PAC-derived cell lines may still represent suitable model systems for an *FGFR2*-targeted approach. Furthermore, canine and human TCC tissues and cell lines have been found to overexpress the RTKs *EGFR* and *ERBB2* [65, 89–91]. This overexpression is thought to be relevant for tumor progression, making *EGFR* a therapeutic target. A first study has shown promising effects of the *EGFR* and *ERBB2* inhibitor lapatinib on canine TCC cell lines [92], while the *EGFR* inhibitor erlotinib reduced proliferation in a subset of human TCCs [93, 94]. *EGFR* and *ERBB2* were not differentially expressed in the present sample set, suggesting that the characterized cell lines are suitable models for *EGFR*-targeted therapies. Furthermore, the tyrosine kinases and therapeutical targets *EPHA2, MET*, *LYN* and *PDGFRA* were expressed by specific cell lines at the same level compared to the average of the tissues. Claudins participate in the formation of tight junctions and are therefore essential for the maintenance of an epithelial structure. To promote EMT and metastasis, tight junctions are dismantled in favor of a more invasive, mesenchymal cell type [43, 95]. Therefore, claudins play an important role in tumor progression and are promising therapeutic targets [43, 96]. The present findings confirm the results of a previous study investigating these four claudins at RNA and protein level in Adcarc1258, Adcarc0846 and TCC0840 [43]. Among PAC cell lines, Adcarc0846 had the highest expression of *CLDN1*, *3*, *4* and *7* and the lowest invasive potential and is hence the most suitable for a claudin-targeted therapy approach. In contrast, Adcarc1258 was highly invasive, expressed all four claudin genes at the lowest level among all cell lines and could therefore be used as negative control.

Correlating molecular marker signatures with outcome or chemoresistance can help unravel resistance mechanisms and identify predictive markers [97] for personalized medicine [18, 98, 99]. ProGENI found a correlation between the G2/M transition of the mitotic cell and resistance to doxorubicin in the nine analyzed cell lines. Consistently, doxorubicin arrests the human PAC cell line DU145 [100] in the G2/M transition and thereby induces apoptosis. Presumably, an altered G2/M transition might now explain the failure of doxorubicin to induce apoptosis in the particularly resistant Metadcarc1511.2 and Metadcarc1511.3 [37]. Furthermore, doxorubicin resistance in cell count was herein associated with a modified phosphatidylserine and phosphoethanolamine biosynthesis. The authors of a study conducted on doxorubicin-resistant MCF7 cells [101] obtained similar results and discussed a less fluid cell membrane as one aspect of chemoresistance. However, it remains to be investigated whether the particular doxorubicin resistance of Metadcarc1511.2 and Metadcarc1511.3 is truly associated with these findings, or whether it is simply a patient-specific feature. Consistent with results of other research groups [102, 103], ProGENI correlated resistance towards platinum drugs with modifications in histone deacetylation. Following this approach, histone deacetylase inhibitors show anti-proliferative effects against canine PAC and TCC cell lines, and might be promising when combined with carboplatin [35, 86, 104]. Additionally, recent studies confirmed binding sites of cisplatin and doxorubicin to tRNA [105, 106]. This supports the observed association of tRNA splicing and the tRNA-intron endonuclease complex with carboplatin and doxorubicin resistance in the characterized nine cell lines. However, this analysis was limited by the relatively small sample size of nine canine cell lines. As next generation sequencing emerges in veterinary medicine, this analysis should be repeated with the transcriptomic and chemosensitivity data of more canine cell lines. Another interesting aspect might be to repeat this analysis, including cell lines selected for doxorubicin or carboplatin resistance.

Last but not least, this analysis is based on the transcriptome and only selected genes were verified on the protein level. Since translation is influenced by multiple regulatory mechanisms, mRNA levels are not unconditionally transferrable to protein levels [107]. Additionally, protein functionality might be affected by mutations and alternative splicing, which was also not the subject of this analysis. Targeted chemotherapy and resistance mechanisms operate on the protein level. Thus, the targets of interest and their functionality warrant further investigation. Furthermore, since the cell lines were examined only at one time-point, this study is unable to make definitive statements concerning their gene expression stability. However, the present study characterized the cell lines beyond passage 60 and the largest changes in cell lines normally occur during the first 30 passages [12]. Hence, a certain stability can be assumed.

In essence, compared to their original tumor tissues, the nine canine cell lines characterized in the present study conserved EMT gene expression programs and downstream PI3K-AKT signaling. Due to their individual therapeutic target signatures, they constitute a suitable panel to evaluate a large variety of inhibitors in vitro. Additionally, the panel includes cell lines derived from primary tumors and metastases of the same patient, which are interesting subjects for metastasis research.

## Conclusions

Enabled by the rare availability of the tissue samples from which they were derived, this study assessed at the transcriptomic level nine canine PAC, PAC metastasis and TCC cell lines as in vitro models for targeted therapies in canine prostate cancer. Adaptation to culturing conditions and clonal selection resulted in the downregulation of genes associated with tumor microenvironment in the cell lines compared to their original tissues, while tumor- and patient-specific characteristics, as well as the extent of EMT, were mostly conserved. Most importantly, all nine cell lines featured unique therapeutic target expression signatures and preserved subsequent signaling cascades. In summary, this dataset constitutes a rich resource for choosing suitable in vitro models for testing future therapeutic approaches for canine prostate cancer.

## Supplementary Information

**Additional file 1.** Functional analysis of differentially expressed genes (DEGs) and categorization of retrieved enriched biological processes.

**Additional file 2.** The ProGENI top 300 genes associated with doxorubicin and carboplatin resistance quantified by metabolic activity and cell count in the characterized cell lines.

**Additional file 3.** Functional analysis of top 300 genes associated with chemoresistance.

**Additional file 4.** Log2 read counts of therapeutically relevant RTK genes.

## Data Availability

The datasets generated and/or analyzed during the current study are available in the Gene Expression Omnibus database, [https://www.ncbi.nlm.nih.gov/geo/query/acc.cgi?acc=GSE190374].

## Abbreviations

PAC: Prostate adenocarcinoma
TCC: Transitional cell carcinoma
MCRPC: Metastatic castration-resistant prostate cancer
EMT: Epithelial to mesenchymal transition
RTK: Receptor tyrosine kinases
P: Prostate
B: Urinary bladder
Ln: Lymph node
Tiho: University of Veterinary Medicine Hannover
D: Dog
Pro: Prostate
Urt: Urinary tract (urinary bladder)
Adcarc: Adenocarcinoma
Carc: Carcinoma
Metadcarc: Metastasis of an adenocarcinoma
Adcarc1258: TihoDProAdcarc1258
Adcarc0846: TihoDProAdcarc0846
Adcarc1508: TihoDProAdcarc1508
Adcarc1511.1: TihoDProAdcarc1511.1
Metadcarc1511.2: TihoDProMetadcarc1511.2
Metadcarc1511.3: TihoDProMetadcarc1511.3
TCC0840: TihoDProCarc/TCC0840
TCC1509: TihoDProTCC1509
TCC1506: TihoDUrtTCC1506
DMSO: Dimethyl sulfoxide
STAR: Spliced Transcripts Alignment to a Reference
DAVID: Database for annotation, visualization and integrated discovery tool
GO: Gene Ontology
BP: Biological process
CC: Cellular component
FDR: False discovery rate
REVIGO: Reduce + visualize gene ontology
KEGG: Kyoto Encyclopedia of Genes and Genomes
ProGENI: Prioritization of Genes Enhanced with Network Information
STRING: Search tool for recurring instances of neighboring genes
GFR: Growth factor receptor
GF: Growth factor
