# Supplementary figures and images for: RNA-seq of nine canine prostate cancer cell lines reveals diverse therapeutic target signatures
Source: Cancer Cell Int. 2022 Feb 2;22:54. doi: 10.1186/s12935-021-02422-9 (PMC8812184; doi:10.1186/s12935-021-02422-9)

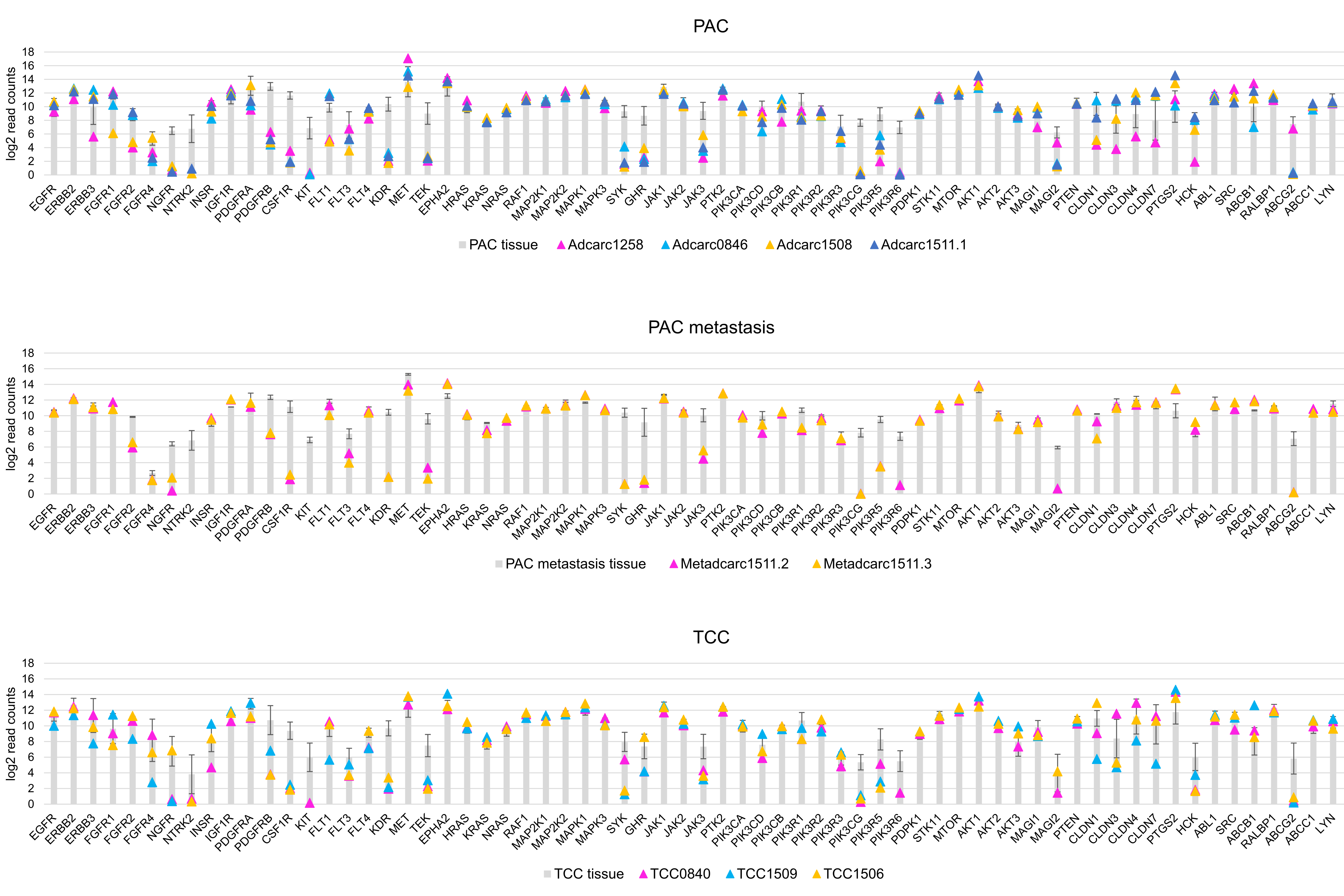

Supplement: Supplementary file 4 — Additional file 4. Log2 read counts of therapeutically relevant RTK genes. [file 12935_2021_2422_MOESM4_ESM.png]
